# The impact of angiotensin-converting-enzyme inhibitors versus angiotensin receptor blockers on 3-year clinical outcomes in patients with acute myocardial infarction without hypertension

**DOI:** 10.1371/journal.pone.0242314

**Published:** 2020-11-30

**Authors:** Ae-Young Her, Byoung Geol Choi, Seung-Woon Rha, Yong Hoon Kim, Cheol Ung Choi, Myung Ho Jeong

**Affiliations:** 1 Division of Cardiology, Department of Internal Medicine, Kangwon National University School of Medicine, Chuncheon, South Korea; 2 Cardiovascular Center, Korea University Guro Hospital, Seoul, South Korea; 3 Department of Medicine, Korea University Graduate School, Seoul, South Korea; 4 The Heart Center of Chonnam National University Hospital, Gwangju, South Korea; East Tennessee State University, UNITED STATES

## Abstract

This study aimed to investigate the impact of angiotensin-converting-enzyme inhibitors (ACEI) and angiotensin II type 1 receptor blockers (ARB) on 3-year clinical outcomes in acute myocardial infarction (AMI) patients without a history of hypertension who underwent successful percutaneous coronary intervention (PCI) with drug-eluting stents (DES). A total of 13,104 AMI patients who were registered in the Korea AMI registry (KAMIR)-National Institutes of Health (NIH) were included in the study. The primary endpoint was 3-year major adverse cardiac events (MACE), which was defined as the composite of all-cause death, recurrent myocardial infarction (MI), and any repeat revascularization. To adjust baseline potential confounders, an inverse probability weighting (IPTW) analysis was performed. The patients were divided into two groups: the ACEI group, n = 4,053 patients and the ARB group, n = 4,107 patients. During the 3-year clinical follow-up, the cumulative incidences of MACE (hazard ratio [HR], 0.843; 95% confidence interval [CI], 0.740–0.960; p = 0.010), any repeat revascularization (HR, 0.856; 95% CI, 0.736–0.995; p = 0.044), stroke (HR, 0.613; 95% CI, 0.417–0.901; p = 0.013), and re-hospitalization due to heart failure (HF) (HR, 0.399; 95% CI, 0.294–0.541; p <0.001) in the ACEI group were significantly lower than in the ARB group. In Korean patients with AMI without a history of hypertension, the use of ACEI was significantly associated with reduced incidences of MACE, any repeat revascularization, stroke, and re-hospitalization due to HF than those with the use of ARB.

## Introduction

Secondary prevention of cardiovascular and cerebrovascular events, which includes optimal medical therapy, is extremely important in patients after acute myocardial infarction (AMI) because they remain at high risk for recurrent ischemic events [1, 2]. Among the available therapies, the current European guidelines recommend that treatment with angiotensin-converting-enzyme inhibitors (ACEI) should be considered in all patients with ST-segment elevation myocardial infarction (STEMI) [3, 4]. ACEI are also indicated in non ST-segment elevation myocardial infarction (NSTEMI) patients with hypertension, systolic left ventricular dysfunction, heart failure (HF), or diabetes [5–7], in the absence of contraindications such as hypotension or severe renal dysfunction. The guidelines also recommend that angiotensin II type 1 receptor blockers (ARB) should be considered as alternatives to ACEI, particularly if patients cannot tolerate ACEI [3, 5].

Although many previous trials have demonstrated the beneficial role of ACEI or ARB in AMI patients and treatment with ACEI or ARB have demonstrated particular benefits in high-risk hypertensive patients [7–10], a recent meta-analysis of several randomized controlled trials showed that among patients with a clinical history of cardiovascular disease without hypertension, treatment with ACEI or ARB significantly reduced the composite cardiovascular events (odds ratio [OR], 0.89; 95% confidence interval [CI], 0.85–0.93) [11]. Moreover, in patients with AMI, the prevalence of prehypertension or normotension varies from 40 to 70% [12, 13]. However, few studies have directly compared trials of ACEI and ARB in AMI patients without hypertension, and there is limited data on the relative superiority or similarity of the effects of ACEI and ARB on the long-term clinical outcomes in these patients.

Therefore, the aim of this study was to investigate the impact of ACEI and ARB on 3-year clinical outcomes in AMI patients without a history of hypertension who underwent successful percutaneous coronary intervention (PCI) with drug-eluting stents (DES).

## Materials and methods

### Study population

**Fig 1:**
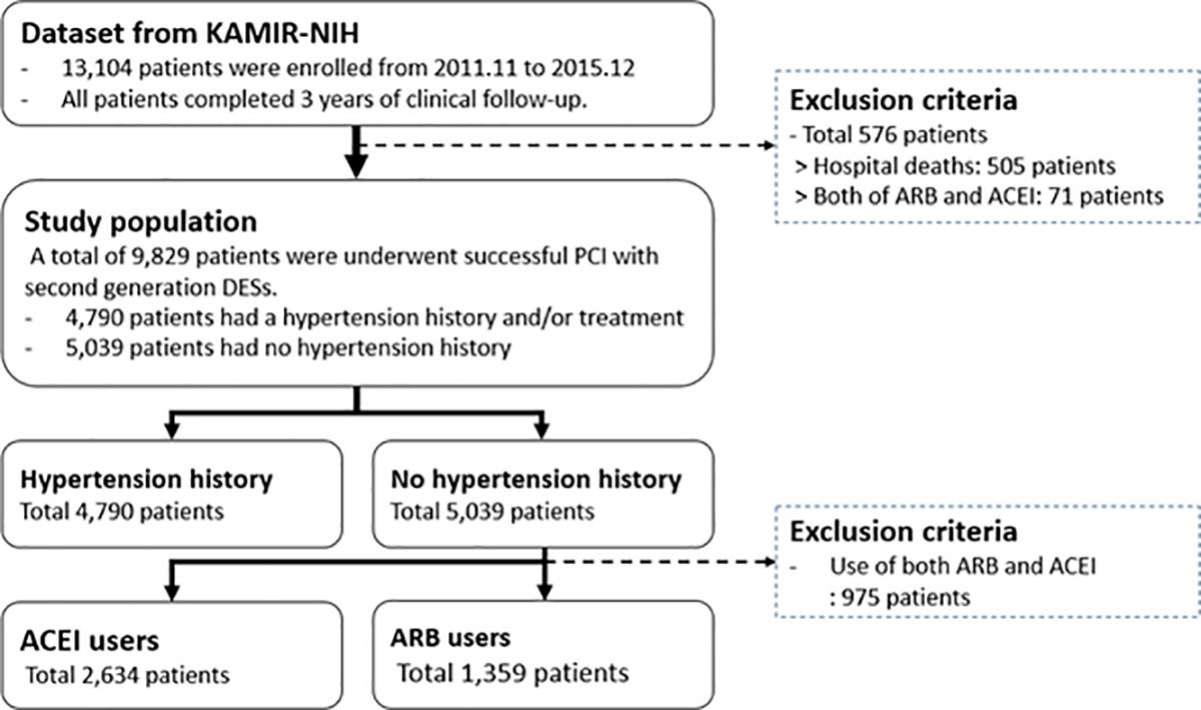
Flow chart of this study.

The study population was enrolled from the Korea AMI registry (KAMIR). The design of the KAMIR study has been described in our previous studies [14, 15]; the details of the registry can be found at the KAMIR website (http://www.kamir.or.kr). Briefly, the KAMIR study is a prospective, multicenter online registry that was designed to reflect the “real world” practice in a series of Korean AMI patients treated since November 2005 wherein their current epidemiology and clinical outcomes were investigated. To accurately reflect the current standard clinical practice in all Korean AMI patients, we selectively analyzed the results from a special qualified KAMIR registry subset named the KAMIR-national institute of health (NIH) registry. From November 2011 to December 2015, a total of 13,104 AMI patients were enrolled in the nationwide KAMIR-NIH registry. The flow chart shows the present study scheme (Fig 1). Of 9,829 patients who underwent successful PCI with second-generation DES, 5,039 patients did not have a history of hypertension. After excluding patients who used combined ACEI and ARB, a total of 3,993 patients who used ACEI or ARB were classified into two groups; the ACEI group (n = 2,634) and the ARB group (n = 1,359). Among two groups, patients with STEMI and NSTEMI at final diagnosis in ACEI group were 1,536 patients (58.3%) and 1,098 patients (41.7%). In ARB group, patients with STEMI and NSTEMI at final diagnosis were 667 patients (49.1%) and 692 patients (50.9%).

All data collection was carried out using the same web-based case report form at each participating center. All patients provided written informed consent prior to enrollment. In this study, all 3,993 patients completed a 3-year clinical follow-up through face-to-face interviews, phone calls, or chart review. This study protocol was approved by the Korea University Guro Hospital Institutional Review Board (IRB) (#2016GR0740) according to the ethical guidelines of the 1975 Declaration of Helsinki.

### PCI procedure and medical treatment

PCI was performed using a standard technique [16]. PCI was initiated through either the femoral or radial artery after administration of a single dose of unfractionated heparin (70–100 U/kg). All patients received loading doses of aspirin (200–300 mg) and other anti-platelet agents, including clopidogrel, ticagrelor, or prasugrel before PCI. DES were deployed after prior balloon angioplasty with or without thrombus aspiration, and the use of anti-coagulation therapy during PCI was left to each physician’s discretion. A successful PCI was defined as the achievement of an angiographic residual stenosis of less than 10% without major adverse cardiac events (MACE) in the presence of a thrombolysis in myocardial infarction blood flow grade 3. The patients maintained dual anti-platelet therapy for at least one year. During the in-hospital period, the patients received medication, including beta-blockers (BB), ACEI, ARB, calcium channel blockers (CCB), and statins. After discharge, the patients were encouraged to maintain the same medication they received in the hospital.

### Study definition and endpoint

The key combined primary endpoint was MACE, which was defined as the composite of all-cause death, recurrent myocardial infarction (MI), and any repeat revascularization. The key secondary endpoints were the occurrence of any clinical events such as all-cause death, recurrent MI, any repeat revascularization including surgical coronary artery bypass graft (CABG) or repeat PCI, target lesion failure, stroke, stent thrombosis, and re-hospitalization due to HF. All deaths were considered cardiac in origin, unless a non-cardiac origin was definitely documented. Recurrent MI was defined as recurrent symptoms with new ST-segment elevation or re-elevation of cardiac markers to at least twice the upper limit of normal, and periprocedural MI was not included as a clinical outcome. Target lesion revascularization (TLR) was defined as repeat PCI within the index procedure stent or 5 mm edge. Target vessel revascularization (TVR) was defined as any repeat PCI or surgical CABG of any segment in the target vessel. Any repeat revascularization was defined as any repeat PCI or CABG of the target vessel or the non-target vessel. Target lesion failure was defined as the composite of clinically driven TLR, recurrent MI, or cardiac death related to the target vessel. All participants were required to visit the outpatient cardiology department at the end of the first month and subsequently every six months after the PCI procedure, as well as whenever angina-like symptoms occurred.

### Statistical analysis

For continuous variables, the differences between the two groups were evaluated using the unpaired t-test or Mann-Whitney rank test. Data were expressed as mean ± standard deviations. For discrete variables, differences between the two groups were expressed as counts and percentages, and analyzed with the χ2 or Fisher’s exact test. To adjust for any potential confounders, an inverse probability weighting (IPTW) analysis was performed using the logistic regression model. We tested all available variables that could be potentially relevant: age, sex (male), body mass index, Killip class on admission, left ventricular ejection fraction, cardiovascular risk factors (e.g., hypertension, dyslipidemia, HF, cerebrovascular disease, prior CABG, prior MI, and prior PCI), co-medication treatment (e.g., aspirin, other anti-platelets, CCB, BB, and statins), and angiographic and procedural characteristics (e.g., target vessel, number of diseased vessels, lesion type, and DES type). Various clinical outcomes up to 3 years were estimated using the Kaplan-Meier analysis, and differences between the groups were compared with the log-rank test before and after IPTW. Binary logistic regression analysis was used to assess the hazard risk (HR) of the ACEI group compared to that of the ARB group in the IPTW population. For all analyses, a two-sided p <0.05 was considered statistically significant. All data were processed with SPSS (version 20.0, SPSS-PC, Inc. Chicago, Illinois).

### Ethical approval

This study protocol was approved by the Korea University Guro Hospital Institutional Review Board (IRB) (#2016GR0740) according to the ethical guidelines of the 1975 Declaration of Helsinki.

## Results

The baseline clinical characteristics of the patients are shown in Table 1. The mean age of the ACEI group was younger than that of the ARB group (59.7 ± 11.8 vs. 60.8 ± 12.2 years; p = 0.004). The mean systolic and diastolic blood pressure (BP) were higher in the ACEI group (systolic BP: 133 ± 26 vs. 129 ± 25 mmHg; p <0.001, diastolic BP: 81 ± 16 vs. 79 ± 16 mmHg; p = 0.013). The incidence of STEMI at final diagnosis was higher in the ACEI group (58.3 vs. 49.1%; p <0.001). The number of patients with a history of MI (4.0 vs. 5.5%; p = 0.027) and PCI (4.6 vs. 6.8%; p = 0.003) were higher in the ARB group. The prevalence of current smoking was higher in the ACEI group (53.2 vs. 48.6%; p = 0.007) and the number of patients with a history of smoking was higher in the ARB group (16.2 vs. 20.8%; p <0.001). However, these intergroup differences in baseline characteristics were well balanced after IPTW adjustment.

**Table 1:** Baseline clinical characteristics.

	Crude population				IPTW			
Variables	ACEI (n = 2,634)	ARB (n = 1,359)	P value	S.diff	ACEI (n = 4,053)	ARB (n = 4,107)	P value	S.diff
Sex, male	2227 (84.5)	1130 (83.1)	0.252	-0.15	3406 (84.0)	3514 (85.6)	0.055	0.17
Age, year	59.7 ± 11.8	60.8 ± 12.2	0.004	0.09	60.3 ± 12.0	59.8 ± 12.2	0.086	-0.04
Blood pressure; BP, mmHg								
Systolic	133 ± 26	129 ± 25	<0.001	-0.14	131 ± 26	132 ± 26	0.142	0.03
Diastolic	81 ± 16	79 ± 16	0.013	-0.08	80 ± 16	81 ± 17	0.088	0.04
Heart rate, beat per minutes	77 ± 17	78 ± 18	0.081	0.06	78 ± 19	78 ± 18	0.663	0.01
Final diagnosis			<0.001	-1.26			0.105	0.24
STEMI	1536 (58.3)	667 (49.1)			2185 (53.9)	2288 (55.7)		
NSTEMI	1098 (41.7)	692 (50.9)			1868 (46.1)	1820 (44.3)		
Killip class								
I	2210 (83.9)	1138 (83.7)	0.893	-0.02	3391 (83.6)	3442 (83.8)	0.862	0.02
II	204 (7.7)	98 (7.2)	0.546	-0.20	288 (7.1)	306 (7.5)	0.549	0.13
III	117 (4.4)	71 (5.2)	0.269	0.36	186 (4.6)	198 (4.8)	0.621	0.11
IV	103 (3.9)	52 (3.8)	0.896	-0.04	188 (4.6)	162 (3.9)	0.121	-0.34
LV ejection fraction, %	51.3 ± 9.9	54.2 ± 10.2	<0.001	0.29	52.2 ± 9.9	51.2 ± 11.7	0.010	-0.09
Clinical history								
Diabetes mellitus	454 (17.2)	261 (19.2)	0.124	0.46	730 (18.0)	726 (17.7)	0.697	-0.08
Dyslipidemia	234 (8.9)	110 (8.1)	0.399	-0.27	345 (8.5)	348 (8.5)	0.952	-0.01
Prior CAD								
Myocardial infarction	105 (4.0)	75 (5.5)	0.027	0.70	231 (5.7)	214 (5.2)	0.332	-0.21
Angina pectoris	121 (4.6)	80 (5.9)	0.077	0.56	224 (5.5)	230 (5.6)	0.883	0.03
Prior PCI	122 (4.6)	93 (6.8)	0.003	0.92	284 (7.0)	257 (6.3)	0.174	-0.29
Prior CABG	7 (0.3)	5 (0.4)	0.576	0.18	13 (0.3)	15 (0.4)	0.731	0.08
Stroke	62 (2.4)	26 (1.9)	0.369	-0.30	88 (2.2)	79 (1.9)	0.429	-0.17
Ischemic	5 (0.2)	3 (0.2)	0.836	0.07	7 (0.2)	8 (0.2)	0.816	0.05
Hemorrhagic	57 (2.2)	23 (1.7)	0.314	-0.34	81 (2.0)	71 (1.7)	0.368	-0.20
Smoking								
Current	1400 (53.2)	661 (48.6)	0.007	-0.63	2104 (51.9)	2141 (52.1)	0.834	0.03
Ex-smoker	424 (16.1)	282 (20.8)	<0.001	1.09	714 (17.6)	718 (17.5)	0.869	-0.03

Data are presented as N (%) or mean ± standard deviation. IPTW: inverse probability weighting; ACEI: angiotensin-converting-enzyme inhibitors; ARB: angiotensin II type 1 receptor blocker; S.diff: standardized mean difference; STEMI: ST-segment elevation myocardial infarction; NSTEMI: non ST-segment elevation myocardial infarction; LV: left ventricular; CAD: coronary artery disease; PCI: percutaneous coronary intervention; CABG: coronary artery bypass graft.

The angiographic and procedural characteristics and medications at discharge are presented in Table 2. The incidence of the left anterior descending artery as the infarct-related artery (51.2 vs. 47.3%; p = 0.020) and that of multi-vessel disease (47.9 vs. 42.2%; p = 0.001) were higher in the ACEI group. The number of patients with the left main coronary artery as the infarct-related artery was higher in the ARB group (1.4 vs. 2.8%; p = 0.002). The Biolimus A9 stent was more frequently deployed in the ARB group (18.5 vs. 24.2%; p <0.001). The mean diameter of the deployed stents was higher in the ACEI group (3.19 vs. 3.16 mm; p = 0.033) and the deployed stents were longer in the ACEI group (29.5 vs. 27.4 mm; p <0.001). The prescription rates of clopidogrel (69.4 vs. 60.4%; p <0.001) and beta-blockers (92.7 vs. 85.7%; p <0.001) were higher in the ACEI group, and those of prasugrel (10.7 vs. 16.0%; p <0.001), ticagrelor (19.1 vs. 22.9%; p = 0.005), and CCB (2.7 vs. 3.9%; p = 0.031) were higher in the ARB group. However, these intergroup differences were well balanced after IPTW adjustment, except for the number and length of stents and the prescription rate of clopidogrel.

**Table 2:** Angiographic, procedural characteristics and medications at discharge.

	Crude population				IPTW			
Variables	ACEI (n = 2,634)	ARB (n = 1,359)	P value	S.diff	ACEI (n = 4,053)	ARB (n = 4,107)	P value	S.diff
**Angiographic and procedural characteristics**								
Infarct-related artery								
LAD	1349 (51.2)	643 (47.3)	0.020	-0.56	2009 (49.6)	2101 (51.2)	0.148	0.23
LCX	403 (15.3)	265 (19.5)	0.001	1.01	708 (17.5)	681 (16.6)	0.286	-0.22
RCA	846 (32.1)	413 (30.4)	0.265	-0.31	1261 (31.1)	1249 (30.4)	0.488	-0.13
Left main	36 (1.4)	38 (2.8)	0.002	0.99	75 (1.9)	76 (1.9)	>0.999	0.00
Multi-vessel disease	1263 (47.9)	574 (42.2)	0.001	-0.85	1851 (45.7)	1913 (46.6)	0.410	0.13
Drug-eluting stents								
Everolimus	1369 (52.0)	691 (50.8)	0.499	-0.16	2118 (52.3)	2102 (51.2)	0.330	-0.15
Zotarolimus	18 (0.7)	14 (1.0)	0.244	0.37	34 (0.8)	41 (1.0)	0.451	0.17
Biolimus A9	487 (18.5)	329 (24.2)	<0.001	1.24	827 (20.4)	869 (21.2)	0.398	0.17
Sirolimus	84 (3.2)	43 (3.2)	0.966	-0.01	125 (3.1)	130 (3.2)	0.833	0.05
Number of stent	1.16 ± 0.40	1.14 ± 0.38	0.151	-0.05	1.15 ± 0.40	1.17 ± 0.44	0.016	0.05
Stent diameter, mm	3.19 ± 0.42	3.16 ± 0.43	0.033	-0.07	3.18 ± 0.42	3.17 ± 0.44	0.428	-0.02
Total stent length, mm	29.5 ± 12.7	27.4 ± 12.2	<0.001	-0.17	28.9 ± 12.4	29.6 ± 13.2	0.016	0.05
**Discharge medication**								
Aspirin	2623 (99.6)	1353 (99.6)	0.913	0.00	4037 (99.6)	4090 (99.6)	0.761	0.00
Clopidogrel	1828 (69.4)	821 (60.4)	<0.001	-1.12	2683 (66.2)	2818 (68.6)	0.020	0.30
Prasugrel	281 (10.7)	218 (16.0)	<0.001	1.47	522 (12.9)	494 (12.0)	0.246	-0.24
Ticargrelor	503 (19.1)	311 (22.9)	0.005	0.83	818 (20.2)	764 (18.6)	0.071	-0.36
Ca-channel blockers	70 (2.7)	53 (3.9)	0.031	0.69	123 (3.0)	138 (3.4)	0.405	0.18
Beta-blockers	2443 (92.7)	1164 (85.7)	<0.001	-0.75	3637 (89.7)	3705 (90.2)	0.474	0.05
Statin	2522 (95.7)	1307 (96.2)	0.521	0.04	3893 (96.0)	3950 (96.2)	0.770	0.01

Data are presented as N (%) or mean ± standard deviation. IPTW: inverse probability weighting; ACEI: angiotensin-converting-enzyme inhibitors; ARB: angiotensin II type 1 receptor blockers; S.diff: standardized mean difference; LAD: left anterior descending artery; LCX: left circumflex artery; RCA: right coronary artery.

**Fig 2:**
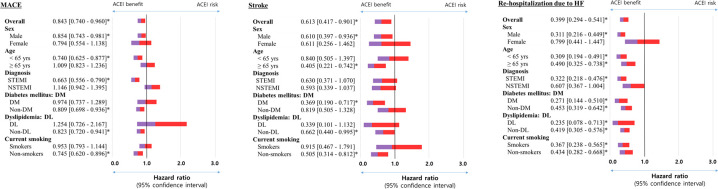
Subgroups analysis for the impact of ACEI compared to that of ARB on (A) MACE, (B) stroke, (C) re-hospitalization due to HF at the 3-year follow-up using binary regression hazard ratio analysis in the IPTW population. The blue and red colors represent the lower and upper limits of 95% CI, respectively. ACEI, angiotensin-converting-enzyme inhibitors; ARB, angiotensin II type 1 receptor blockers; MACE, major adverse cardiac events; HF, heart failure; IPTW, inverse probability weighting.

Table 3 shows the cumulative incidences of major clinical outcomes during the 3-year follow-up period. Before the adjustment, the cumulative incidence of stroke was significantly higher in the ARB group (1.1 vs. 1.8%; p = 0.042). After IPTW adjustment, the cumulative incidences of MACE (hazard ratio [HR], 0.843; 95% CI, 0.740–0.960; p = 0.010), any repeat revascularization (HR, 0.856; 95% CI, 0.736–0.995; p = 0.044), stroke (HR, 0.613; 95% CI, 0.417–0.901; p = 0.013), and re-hospitalization due to HF (HR, 0.399; 95% CI, 0.294–0.541; p <0.001) in the ACEI group were significantly lower than in the ARB group. However, the cumulative incidences of all-cause death, recurrent MI, target lesion failure, and stent thrombosis were similar between the two groups after IPTW adjustment. Fig 2A shows that in cases of male, younger age (<65 years), STEMI, no history of diabetes and dyslipidemia, or non-smoker, ACEI provided greater benefits than did ARB in reducing MACE. In cases of male, older age (≥65 years), history of diabetes, no history of dyslipidemia, or non-smoker, ACEI was found to be more beneficial in reducing stroke compared to ARB (Fig 2B). Fig 2C shows that in cases of male, STEMI or irrespective of age, diabetes, dyslipidemia, and smoking history, ACEI was found to be more beneficial than ARB in reducing re-hospitalization due to HF (Fig 2C).

**Table 3:** Major clinical outcomes after acute myocardial infarction at 3 years.

	Crude population			IPTW				
Variables	ACEI (n = 2,634)	ARB (n = 1,359)	P value	ACEI (n = 4,053)	ARB (n = 4,107)	P value	Hazard ratio [95% CI: Low—High]	P value
MACE	303 (11.5)	159 (11.7)	0.854	486 (12.0)	571 (13.9)	0.010	0.843 [0.740–0.960]	0.010
All-cause death	99 (3.8)	58 (4.3)	0.433	144 (3.6)	161 (3.9)	0.382	0.903 [0.718–1.135]	0.384
Cardiac death	56 (2.1)	32 (2.4)	0.641	82 (2.0)	94 (2.3)	0.409	0.885 [0.656–1.195]	0.427
Non-cardiac death	43 (1.6)	26 (1.9)	0.519	62 (1.5)	68 (1.7)	0.651	0.931 [0.658–1.318]	0.691
Myocardial infarction	47 (1.8)	36 (2.6)	0.070	98 (2.4)	86 (2.1)	0.325	1.148 [0.857–1.539]	0.353
STEMI	11 (0.4)	7 (0.5)	0.663	17 (0.4)	18 (0.4)	0.896	0.953 [0.488–1.862]	0.890
NSTEMI	36 (1.4)	29 (2.1)	0.069	81 (2.0)	69 (1.7)	0.284	1.198 [0.866–1.658]	0.273
Revascularization	206 (7.8)	96 (7.1)	0.391	345 (8.5)	403 (9.8)	0.042	0.856 [0.736–0.995]	0.044
CABG	7 (0.3)	4 (0.3)	0.870	10 (0.2)	11 (0.3)	0.850	0.919 [0.388–2.175]	0.848
PCI	200 (7.6)	93 (6.8)	0.389	337 (8.3)	397 (9.7)	0.033	0.848 [0.728–0.987]	0.034
TLR	52 (2.0)	28 (2.1)	0.854	89 (2.2)	79 (1.9)	0.387	1.138 [0.839–1.546]	0.404
TVR	93 (3.5)	48 (3.5)	0.998	148 (3.7)	136 (3.3)	0.402	1.110 [0.875–1.407]	0.388
Target lesion failure	107 (4.1)	59 (4.3)	0.675	170 (4.2)	171 (4.2)	0.946	1.006 [0.810–1.250]	0.953
Stent thrombosis	10 (0.4)	5 (0.4)	0.954	17 (0.4)	12 (0.3)	0.334	1.443 [0.690–3.017]	0.330
Stroke	28 (1.1)	25 (1.8)	0.042	42 (1.0)	70 (1.7)	0.010	0.613 [0.417–0.901]	0.013
Re-hospitalization due to HF	44 (1.7)	31 (2.3)	0.178	59 (1.5)	148 (3.6)	<0.001	0.399 [0.294–0.541]	<0.001

Data are presented as incidence (%). IPTW: inverse probability weighting, ACEI: angiotensin-converting-enzyme inhibitors; ARB: angiotensin II type 1 receptor blocker; CI: confidence interval; MACE: major adverse cardiac events; STEMI: ST-segment elevation myocardial infarction; NSTEMI: non ST-segment elevation myocardial infarction; CABG: coronary artery bypass graft; PCI: percutaneous coronary intervention; TLR: target lesion revascularization; TVR: target vessel revascularization; HF: heart failure.

## Discussion

The main findings from this study of AMI patients without a history of hypertension who underwent successful PCI with DES over a 3-year follow-up are: 1) the cumulative incidences of MACE, any repeat revascularization, stroke, and re-hospitalization due to HF were significantly lower in the ACEI group than in the ARB group; 2) the cumulative incidences of all-cause death, recurrent MI, target lesion failure, and stent thrombosis were similar between the two groups.

It is well known that renin-angiotensin-aldosterone system (RAAS) inhibitors such as ACEI and ARB reduce the risk of major cardiovascular events in patients with coronary artery disease (CAD) or those who are at a high risk for cardiovascular disease [17, 18]. The current European guidelines recommend that ACEI should be used as the first-line choice among RAAS inhibitors after AMI; ARB should be considered in patients who cannot tolerate ACEI [3, 5]. Moreover, Asian patients, including Koreans, often develop adverse side effects such as cough when they are prescribed ACEI; therefore, many physicians switch from ACEI to ARB assuming that the efficacy of both are comparable [19]. However, there is considerable confusion about the relative efficacy of ACEI and ARB regarding the long-term clinical outcomes in patients with AMI. The Valsartan in Acute Myocardial Infarction (VALIANT) study showed that ARB was as effective as ACEI in reducing the incidence of death, MI, angina, revascularization, or stroke over a 2-year follow-up period (HR, 0.97; 95% CI, 0.91–1.03; p = 0.286) [8]. Another Korean study also showed that ACEI and ARB had similar risks of cardiac death during a 1-year follow-up in AMI patients [20]. However, Hara et al. demonstrated that ACEI was associated with better survival than ARB in AMI patients in the period from 2 to 5 years after AMI (HR, 0.53; 95% CI, 0.38–0.74; p <0.001); the prognostic impact of ACEI and ARB were similar in the first 2 years [21]. Other Korean national registry data also suggested that ACEI had a greater ability to reduce the occurrences of MACE, any repeat revascularization, and TVR compared to ARB in NSTEMI patients with preserved left ventricular systolic function or diabetes during a 2-year follow-up period [15, 22]. In our study, the cumulative incidences of MACE (HR, 0.843; 95% confidence interval [CI], 0.740–0.960; p = 0.010) and any repeat revascularization (HR, 0.856; 95% CI, 0.736–0.995; p = 0.044) in the ACEI group were significantly lower than in the ARB group during the 3-year follow-up period. Although the present study did not show a significant difference in the incidences of death and MI between ACEI and ARB, the results were mostly consistent with those of other previous reports of relatively long-term (at least a 2-year follow-up period) differential clinical outcomes between ACEI and ARB. These controversial results comparing ACEI and ARB for clinical outcomes in patients after AMI may be influenced by the relatively long-term follow-up period. Therefore, well-designed randomized trials with large- scale studies aiming for long-term follow-up are needed to confirm these findings.

ACEI have been shown to have some beneficial effects on cardiovascular outcomes such as AMI or HF through enhancement of endothelial function, cardiovascular remodeling, and the progression of atherosclerosis [23, 24]. Although ARB have many of the same clinical benefits as ACEI, ARB increase the circulating angiotensin II levels through unopposed stimulation of the angiotensin II type 2 receptor. Furthermore, activation of angiotensin II type 2 receptor leads to plaque instability and thrombus formation [18]. In contrast, ACEI suppress angiotensin II, thereby preventing the pathological effects; however, they also prevent the breakdown of bradykinin, thereby inducing additional cardio-protective effects. These processes may explain the superiority of ACEI over ARB in reducing the incidences of MACE, repeat revascularization, stroke, and re-hospitalization due to HF in patients with AMI.

Another important finding of the present study was the confirmation of the clinical benefit of RAAS inhibitors in AMI patients without a history of hypertension. In patients without hypertension, the challenge is finding evidence of the impact of even small changes in blood pressure on vascular events. A meta-analysis was performed of the following ACEI versus placebo trials: the vascular disease including Heart Outcomes Prevention Evaluation (HOPE), European trial on Reduction Of cardiac events with Perindopril in patients with stable coronary Artery disease (EUROPA), and Prevention of Events with Angiotensin-Converting Enzyme Inhibition (PEACE) trials. In all these trials, the initial mean blood pressures were within the normal range (133/79 to 139/78 mmHg) and fell by only a mean of 3/1.5 to 5/3 mmHg, yet cardiovascular mortality was reduced by 17.4% (p <0.01) [4, 25–27]. Another meta-analysis of 162,341 patients showed that ACEI reduced the risk of CAD independent of the effects of lowering blood pressure; moreover, for any given reduction in blood pressure, ACEI reduced the risk of MI and death an additional 15% (p = 0.002) compared to ARB [18, 28, 29]. In the Valsartan Antihypertensive Long-term Use Evaluation (VALUE) trial, the predicted odds ratio for MI was 0.98 for a systolic blood pressure gradient of 2.2 mmHg compared with the observed 1.19 (p = 0.03); the results of ARB-based treatment were worse than predicted from the gradient in the achieved systolic blood pressure with regards to MI [30]. These results are remarkably consistent with our results in that ACEI was associated with a reduced incidence of clinical outcomes than ARB in AMI patients without a history of hypertension. Moreover, the prevalence of prehypertension or normotension varies from 40 to 70% in AMI patients [12, 13], and the prevalence of AMI patients without a history of hypertension was 51% in our registry data. As direct comparison trials of ACEI and ARB in AMI patients without hypertension are scarce, the results of the present study may be particularly meaningful.

The present study has some limitations. First, because this study was conducted on a multicenter national prospective registry, considerable variations were seen in the baseline characteristics of the enrolled patients. However, we performed an adjustment with IPTW analysis to exclude the confounding factors, and, thus, attempted to show the outcomes of real clinical practices in AMI patients. The use of observational registry data from the real-world setting may have provided additional information that could not be obtained from randomized controlled trials. Therefore, these results are expected to contribute to the precise establishment of better management and prevention in patients with AMI. Second, the clinical impact of ACEI or ARB were compared based on the patients’ medications at discharge. In addition, the dose of medications, long-term adherence, discontinuation, and incidence of adverse events were not available in this study. Finally, because this study population was composed of a single race of Korean patients, our findings cannot be applied to patients of other races and ethnic groups. Further studies in varied patient populations are needed to determine the effect of race and ethnicity on these factors.

In conclusion, the use of ACEI in AMI patients without a history of hypertension undergoing PCI with contemporary DES was associated with reduced cumulative incidences of MACE, any repeat revascularization, stroke, and re-hospitalization due to HF at 3 years than those with the use of ARB.
